# Controlled Tensioning Medial Patellofemoral Ligament Augmentation Using Double‐Tape Suture and Arthroscopic Lateral Soft‐Tissue Release for Patellar Instability

**DOI:** 10.1002/atn2.70316

**Published:** 2026-11-12

**Authors:** Hao‐Yang Tan, Yu‐Fan Luo, Jin‐Hao Zeng, Peng Huang, Shuai Jin, Xing‐Hao Deng, Zheng‐Zheng Zhang, Nuo‐Heng Lin, Ai‐Yao Zhou, Zhong Chen

**Affiliations:** ^1^ Department of Orthopedics, Sun Yat‐sen Memorial Hospital Sun Yat‐sen University Guangzhou Guangdong P. R. China; ^2^ Zhongshan School of Medicine Sun Yat‐sen University Guangzhou Guangdong P. R. China; ^3^ Department of Hand Surgery Shenzhen Hospital of Integrated Traditional Chinese and Western Medicine Shenzhen P. R. China; ^4^ Xinhui District Traditional Chinese Medicine Hospital Jiangmen Guangdong P. R. China; ^5^ Yueqing No. 3 People's Hospital Yueqing Zhejiang P. R. China

## Abstract

Overtensioning the medial patellofemoral ligament during reconstruction is a key cause of patellofemoral overload, anterior knee pain, and limited flexion. We present a reproducible, tension‐calibrated augmentation technique using a single 4.75‐mm SwiveLock anchor preloaded with double FiberTape and sutures—eliminating graft harvest. This technique is intended as an augmentation rather than a full ligament substitution. The staged fixation strategy, combined with arthroscopic evaluation and selective lateral release, serves to minimize the risk of medial overconstraint. The anchor is placed at the Schöttle point, and sutures are passed subcapsularly to the medial patella, exiting through two 2.5‐mm transverse bone tunnels. Initial lateral knot fixation is performed, followed by stepwise lateral release and vastus lateralis detachment to relieve residual lateral tension. Final FiberTape fixation is completed under arthroscopic visualization, allowing real‐time adjustment to avoid overtightening. Final fixation was performed at approximately 30° of knee flexion, with the patella centered within the trochlear groove. This technique restores patellar tracking, permits full flexion, and may help reduce the risk of recurrence in recurrent patellar instability.

## Graphical abstract

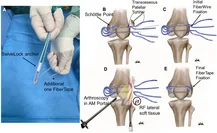

**VIDEO 1:** The procedure was conducted under general anesthesia with the patient in a supine position. The left lower extremity was prepared and draped in the usual sterile fashion. **Step 1: SwiveLock Anchor Preparation With Dual FiberTape Configuration**. The SwiveLock anchor is preloaded with 2 FiberTape sutures, forming 4 reinforcing limbs and 2 control sutures, and set aside for femoral insertion. **Step 2: SwiveLock Anchor Inserted at Schöttle Point for Medial Patellofemoral Ligament (MPFL) Reinforcement**. A 2‐cm incision is made to expose the MPFL femoral footprint, where a bone socket is drilled, and the SwiveLock anchor with 4 FiberTape limbs and 2 FiberWire sutures is inserted for later fixation. **Step 3:** **Patellar MPFL Insertion: Creation of 2 Parallel 2.5‐mm Bone Tunnels for Suture Passage**. Two parallel 2.5‐mm bone tunnels are created at the medial proximal‐to‐mid one‐third junction of the patella—the anatomic MPFL insertion—and 2 passing sutures are placed for suture guidance. **Step 4: Medial Suture Bundle Shuttling via Polydioxanone Along Joint Capsule Surface**. Two suture bundles are shuttled to the medial patellar border using preplaced polydioxanone sutures along the medial joint capsule surface. **Step 5: Suture Passage Through Patellar Tunnels Using Reserved Passing Sutures**. Using the prepositioned passing sutures reserved within the patellar bone tunnels, each suture bundle is smoothly shuttled from the medial side through the tunnels and exits laterally. **Step 6: Initial Lateral Fixation and Medial Tension Assessment**. Initial fixation is achieved by tying the FiberWire sutures laterally, followed by arthroscopic confirmation of medial tension and proper patellar alignment. **Step 7: Arthroscopic Assessment of Medial Tension and Persistent Lateral Tightness**. After preliminary fixation, an arthroscopic view shows the reinforced medial capsule. A probe is used to assess the surface tension, showing clearly tightened soft tissue after FiberTape augmentation. Meanwhile, the lateral retinaculum—marked by the white asterisk (*)—remains under high tension, suggesting persistent lateral soft‐tissue tightness. **Step 8: Lateral Retinacular Release Relieves Soft‐Tissue Tension and Compression**. After lateral retinacular release, the area marked by the asterisk shows clear relaxation of the lateral retinaculum and reduced patellofemoral compression within the dashed lines. **Step 9: Vastus Lateralis Release Restores Patellofemoral Alignment and Reduces Tilt**. After vastus lateralis release, the asterisked region shows restored patellofemoral alignment, resolving lateral tilt and confirming pressure relief. **Step 10: Final FiberTape Fixation Ensures Balanced Patellar Tracking**. Subsequently, final FiberTape fixation is performed under arthroscopic guidance to ensure balanced patellar tracking without overtension. **Step 11: Full Range of Motion Achieved With Stable Patella and No Overload**. At the end of the procedure, full range of motion is achieved, with stable patellar tracking and no signs of dislocation or patellofemoral joint overload. Video content can be viewed at https://doi.org/10.1002/atn2.70316.

Recurrent patellar instability is frequently linked to medial patellofemoral ligament (MPFL) insufficiency, which serves as the primary soft‐tissue restraint against lateral patellar displacement during early knee flexion. MPFL reconstruction has become a widely accepted intervention for symptomatic instability, with favorable mid‐ to long‐term outcomes in restoring patellar tracking and minimizing recurrence of dislocation.^1^, ^2^, ^3^, ^4^

One of the major technical challenges in MPFL reconstruction lies in achieving optimal graft tension. Excessive tension may increase medial patellofemoral joint contact pressures, resulting in anterior knee pain, limited flexion, or chondral overload.^5^ In contrast, insufficient tension can lead to inadequate medial stabilization, causing persistent instability or residual apprehension.^6^

Conventional techniques typically rely on autograft or allograft tendons (e.g., gracilis or semitendinosus), combined with multiple fixation devices. These methods introduce potential downsides, including donor‐site morbidity, prolonged surgical time, and limited intraoperative adjustability.^7^, ^8^ This technique allows intraoperative tension modulation rather than true adjustable fixation systems such as suspensory devices. Patellar fixation, especially with large tunnels or rigid hardware, may further increase the risk of patellar fracture.^9^

To address these concerns, we describe a tension‐controlled MPFL augmentation technique using a single 4.75‐mm biocomposite SwiveLock (Arthrex, Naples, FL) anchor preloaded with dual FiberTape (Arthrex, Naples, FL) sutures. This method avoids graft harvesting and achieves medial reinforcement via two 2.5‐mm transverse patellar tunnels at the anatomic MPFL insertion. High‐strength sutures are used for initial tensioning, followed by final controlled fixation under arthroscopic guidance.

A unique aspect of this approach is the stepwise release of the lateral retinaculum and vastus lateralis before securing the FiberTape. This maneuver permits real‐time arthroscopic assessment of patellofemoral congruence and dynamic tracking, allowing for precise modulation of medial restraint. Such controlled tensioning helps to prevent overconstraint and patellofemoral overload, while promoting better alignment of the quadriceps vector.^10^, ^11^ Lateral release was performed selectively in patients with persistent lateral tightness, with care taken to avoid iatrogenic instability.

Overall, this technique offers a graft‐free, tension‐adjustable, and anatomically guided solution for patellar instability, providing staged tension control rather than true adjustable fixation. It prioritizes functional restoration and real‐time soft‐tissue balancing to improve joint biomechanics.

## SURGICAL TECHNIQUE

Controlled tensioning MPFL augmentation and arthroscopic lateral soft‐tissue release are described step‐by‐step below and are shown in Video 1. Pearls and pitfalls are presented in Table 1.

**TABLE 1:** Pearls and Pitfalls

**Pearls**	**Pitfalls**
Dual FiberTape configuration secured with a single SwiveLock provides strong medial reinforcement with balanced load distribution.	Misplacement of patellar tunnels may cause cortical breach or stress risers, increasing risk of fracture.
Two anatomically placed 2.5‐mm patellar bone tunnels offer stable fixation while minimizing the risk of iatrogenic patellar fracture.	Tunnels that are too central or superficial may compromise structural integrity.
FiberWire limbs allow robust preliminary tensioning and enable real‐time assessment of patellar tracking under arthroscopy.	Skipping arthroscopic verification can result in overtensioning or insufficient stabilization.
Final FiberTape fixation is performed under arthroscopic guidance, ensuring optimal patellofemoral alignment.	

### Step 1: Anchor Preparation

A 4.75‐mm biocomposite SwiveLock anchor is preloaded with 1 original FiberTape, 1 original FiberWire (Arthrex, Naples, FL), and an additional FiberTape (Figure 1A), producing 4 FiberTape limbs and 2 FiberWire limbs. These 6 strands are evenly split into 2 bundles—each with 2 FiberTape limbs and 1 FiberWire—for later patellar passage. The assembled anchor is reserved for femoral insertion.

**FIGURE 1:**
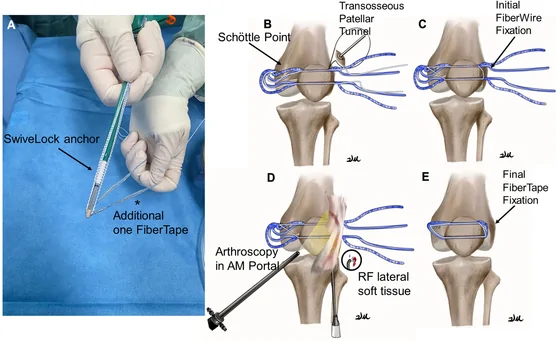
Stepwise construction of a controlled medial patellar restraint using a SwiveLock anchor and suture tape. (A) A 4.75‐mm SwiveLock anchor (Arthrex, Naples, FL) is preloaded with 1 original FiberTape, 1 original FiberWire, and an additional FiberTape (*), which produces 4 FiberTape limbs and 2 FiberWire limbs. (B) Preloaded SwiveLock anchor is inserted into the drilled 4.5‐mm socket determined by the Schöttle point (1 mm anterior to the posterior cortex extension line, 2.5 mm distal to the perpendicular line from the posterior medial condyle, and proximal to the Blumensaat line). The 2 suture bundles are routed subcutaneously along the medial capsule and passed from medial to lateral through the 2 parallel 2.5‐mm transosseous patellar tunnels, 1 in the upper half of the patella, and the other at the junction of its proximal and middle thirds. (C) Initial stabilization is achieved by tensioning and tying the 2 FiberWire sutures over the lateral patellar cortex. However, 4 FiberTape sutures remain untied to facilitate subsequent fixation. (D) A lateral retinacular release and vastus lateralis release via radiofrequency are performed under arthroscopy in the anteromedial portal before final fixation. (E) The 4 FiberTape limbs are tied in matched pairs over the lateral patellar cortex under arthroscopy. (AM, anteromedial; RF, radiofrequency.)

### Step 2: Femoral Tunnel Preparation and Anchor Insertion

A 2‐cm longitudinal incision is made at the anatomic MPFL femoral footprint, identified via the Schöttle point: 1 mm anterior to the posterior cortex extension line, 2.5 mm distal to the perpendicular line from the posterior medial condyle, and proximal to the Blumensaat line. After splitting the adductor tendon, a 4.5‐mm socket is drilled, and the preloaded SwiveLock anchor is inserted (Figure 2A), with all 6 sutures reserved for medial routing (Figure 2B).

**FIGURE 2:**
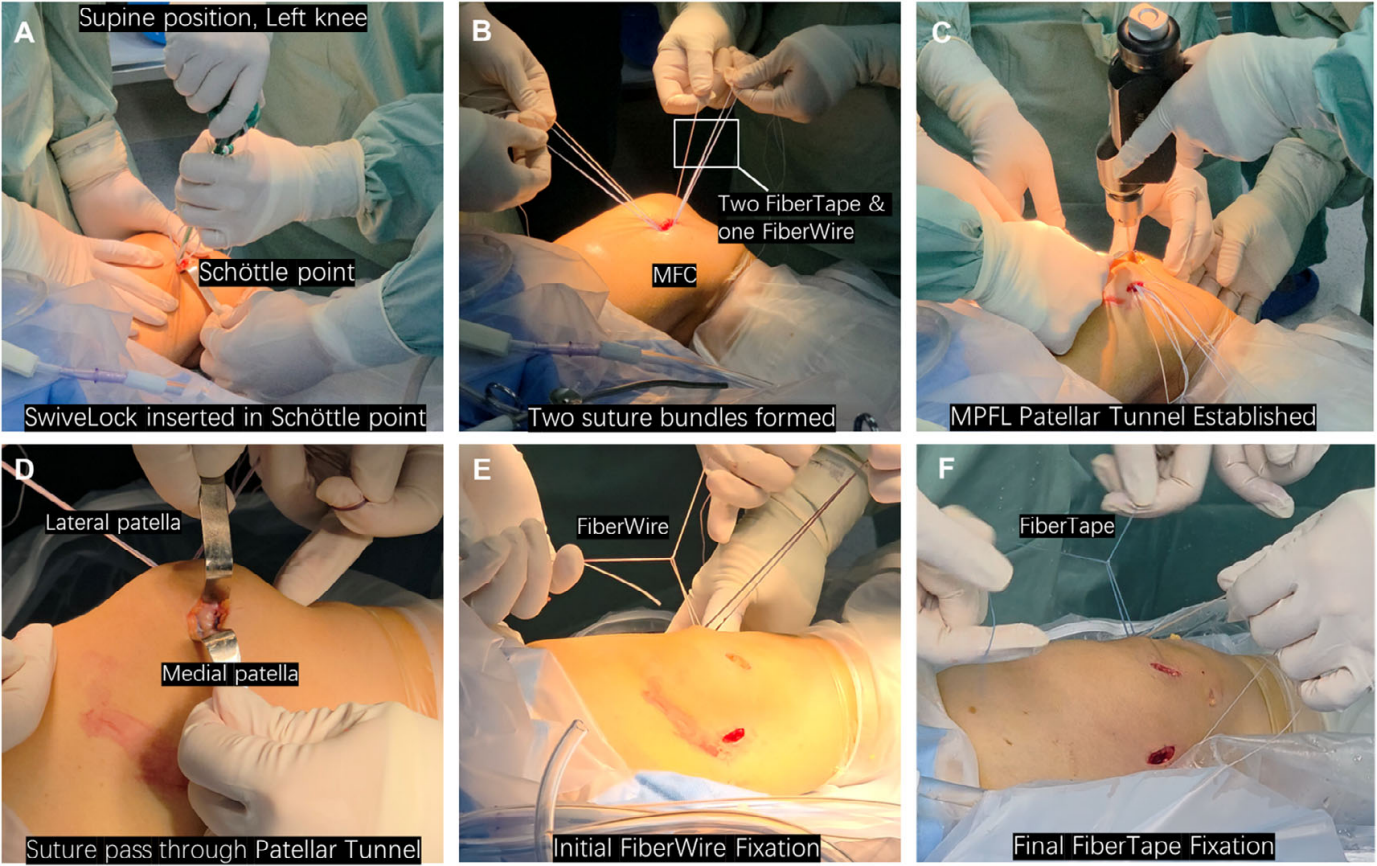
Surgical steps. The procedure was conducted with the patient in a supine position. The left lower extremity was prepared and draped in the usual sterile fashion. (A) Insertion of the preloaded SwiveLock anchor into the anatomical MPFL femoral footprint, located just anterior and distal to the adductor tubercle at the Schöttle point. (B) After anchor insertion into the femoral MPFL footprint, 2 evenly distributed suture bundles are formed—each comprising 2 FiberTape limbs and 1 FiberWire suture. (C) Two parallel 2.5‐mm transverse tunnels are drilled at the superomedial third of the patella, corresponding to the anatomic insertion zone of the MPFL. (D) Subsynovial passage of both suture bundles along the medial surface of the joint capsule toward the patella. (E) Preliminary fixation is achieved by tying 2 FiberWire sutures (1 from each suture bundle) together. (F) Final FiberTape fixation is achieved by tying the 4 FiberTape limbs in matched pairs under arthroscopic visualization. (MFC, medial femoral condyle; MPFL, medial patellofemoral ligament.)

### Step 3: Patellar Tunnel Creation and Suture Passage

Two parallel 2.5‐mm transverse tunnels are drilled in the upper half of the patella, at the junction of the proximal and middle thirds (Figure 2C). This positioning optimizes anatomical fixation and minimizes fracture risk. The 2 suture bundles are routed subcutaneously along the medial capsule and passed from medial to lateral through the tunnels using 1‐0 polydioxanone passing sutures. As shown in Figure 2D, the bundles exit laterally, confirming proper positioning and passage (Figure 1B).

### Step 4: Initial Fixation and Intra‐articular Assessment

Initial stabilization is achieved by tying the 2 FiberWire sutures over the lateral patellar cortex (Figures 1C and 2E). These sutures can be tensioned securely without immediate overcorrection because of the restraining effect of the intact lateral retinaculum. Arthroscopic evaluation confirms medial capsule reinforcement, visible as FiberTape tension just beneath the synovium (Figure 3A). However, excessive tension within the lateral gutter persists because of the intact lateral retinaculum (Figure 3B), indicating the need for additional release.

**FIGURE 3:**
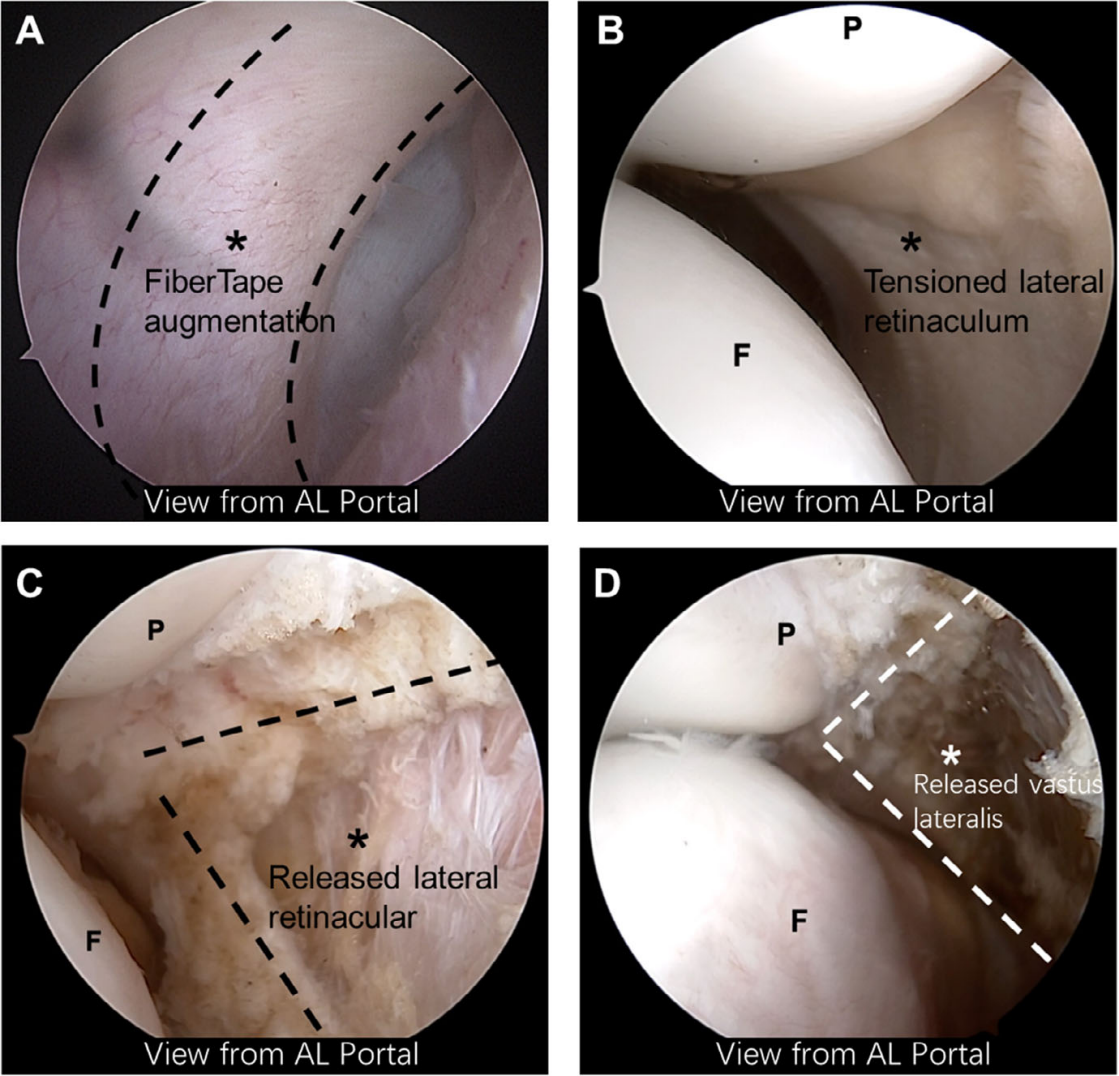
Arthroscopic intra‐articular assessment. (A) Arthroscopic view from anterolateral portal showing reinforced medial retinaculum (*) under FiberTape tension, indicating effective medial reinforcement. (B) Under arthroscopy, smooth lateral patellofemoral articulation with no evidence of lateral subluxation. The asterisk (*) indicates the lateral recess capsule. (C) Lateral retinacular release was completed under the guidance of arthroscopy (*), as evidenced by decompressed lateral retinacular fibers (dashed lines), improving patellar lateral glide. (D) Arthroscopic release of the vastus lateralis muscle head (*), reducing lateral compressive forces and enhancing proximal patellar mobility; white dashed lines delineate the muscle‐tendon junction. (AL: anterolateral; F, femur; P, patella.)

### Step 5: Lateral Release and Final Fixation

To optimize patellar tracking and avoid flexion deficits, a lateral retinacular release (Figure 3C) and vastus lateralis release (Figure 3D) are performed before final fixation (Figure 1D). The extent of release was determined arthroscopically based on the restoration of patellar centralization and reduction of lateral compartment compression. This reduces lateral vector tension and facilitates quadriceps realignment. Arthroscopy confirms improved patellofemoral congruity and appropriate medial reinforcement. Under arthroscopy, the 4 FiberTape limbs are tied in matched pairs over the lateral patellar cortex (Figures 1E and 2F). No symptomatic knot irritation was observed; however, careful knot placement and soft‐tissue coverage are recommended. This staged, arthroscopically guided fixation permits real‐time modulation of medial force, preventing overtightening and preserving full range of motion. During this process, fixation at approximately 30° of knee flexion with arthroscopic confirmation was used to minimize the risk of overtightening. The result is a balanced construct that restores patellar stability while minimizing patellofemoral joint overload.

### Postoperative Rehabilitation

Rehabilitation begins immediately with protection of the construct and early motion. A knee brace locked in extension is used for ambulation for 2 weeks, while passive range of motion starts on postoperative day 1, progressing to 90° by 2 weeks and full motion by 4 to 6 weeks. Partial weight‐bearing is allowed and advanced to full by 4 weeks. Early quadriceps activation is encouraged. After 6 weeks, strengthening and neuromuscular training are introduced, with return to running and sports at 3 to 4 months once strength and control are restored. Excessive lateral stress is avoided during early recovery to maintain patellofemoral balance.

## DISCUSSION

Patellar instability remains a complex clinical challenge, particularly in cases of recurrent dislocation or maltracking. MPFL reconstruction is the cornerstone surgical solution, because the MPFL serves as the primary soft‐tissue restraint to lateral patellar translation.^3^ However, traditional reconstruction techniques involving tendon grafts are often associated with complications such as graft overtensioning, tunnel malposition, and postoperative stiffness.^12^ To address these limitations, the tension‐controlled MPFL augmentation technique using dual FiberTape under arthroscopic guidance offers a reproducible and dynamically adjustable alternative (Table 2). In addition to eliminating graft morbidity, the technique allows staged tension control and intraoperative dynamic assessment.

**TABLE 2:** Advantages and Disadvantages

**Advantages**	**Disadvantages**
Controlled tensioning minimizes risk of overtightening and patellofemoral joint overload.	Requires arthroscopic equipment and skills, increasing surgical complexity and time.
Dual FiberTape with 2 patellar tunnels offers robust and evenly distributed medial support.	Improper tunnel placement may risk patellar fracture or uneven tension distribution.
Preliminary fixation allows intraoperative tension adjustment and dynamic tracking assessment.	Inappropriate lateral release may cause excessive patellar laxity or muscle imbalance.
Arthroscopic final fixation ensures precise patellar alignment under visual guidance.	

Conventional methods commonly use autografts or allografts—typically gracilis or semitendinosus tendons—fixed at both the femoral and patellar sites using anchors or screws. Although these biological grafts are familiar to surgeons, they introduce donor site morbidity, prolonged surgical time, and potential for imprecise tensioning.^13^ Biomechanically, tendon grafts are prone to elongation under cyclic load, and their strength degrades during biological remodeling, potentially contributing to early loosening.^14^ In contrast, FiberTape shows minimal elongation and sustained mechanical strength throughout the range of motion.^15^ The low elongation of FiberTape requires precise tunnel placement and careful tensioning, as there is limited margin for error. Moreover, most traditional techniques lack intraoperative feedback mechanisms to assess patellar tracking in real time, limiting the surgeon's ability to fine‐tune medial‐lateral balance.^16^

This approach leverages the high tensile strength and durability of synthetic suture tape, eliminating the need for graft harvest. The use of a single SwiveLock anchor loaded with 2 FiberTape limbs provides robust medial reinforcement. The creation of dual patellar tunnels distributes medializing forces more evenly, reducing stress concentration and minimizing the risk of tunnel widening or patellar fracture.^17^

A key advantage is the stepwise fixation strategy: preliminary fixation is achieved by tying high‐strength sutures (FiberWire) laterally, allowing the surgeon to assess patellar tracking arthroscopically before the final FiberTape fixation. This real‐time tension modulation helps avoid overcorrection that may result in patellofemoral hypercompression or limited flexion.^12^, ^18^ Arthroscopic visualization ensures that the medial tension is sufficient for stabilization without excessive constraint.

In cases with persistent lateral tightness, lateral retinacular and vastus lateralis release was performed after provisional fixation. These adjunct procedures are especially valuable in patients with strong lateral vector forces, improving soft‐tissue balance and facilitating quadriceps realignment.^19^ Together, they enhance patellar tracking, reduce recurrence, and support better postoperative range of motion.^20^

Despite its advantages, the technique does require arthroscopic proficiency and suture handling skills, presenting a potential learning curve. Additionally, while synthetic implants provide mechanical durability, they do not integrate biologically, and their long‐term performance under repetitive stress remains under investigation. Nonetheless, early outcomes indicate satisfactory patellar tracking, improved stability, and preserved flexion.

In summary, this tension‐controlled, graft‐free MPFL augmentation technique offers a precise, dynamically adjustable, and reproducible solution for patellar instability. By avoiding graft harvest, allowing intraoperative tension assessment, and incorporating soft‐tissue balancing, it represents a promising alternative to conventional reconstruction strategies.

## DISCLOSURES

The authors (H‐Y.T., Y‐F.L., J‐H.Z., P.H., S.J., X‐H.D., Z‐Z.Z., N‐H.L., A‐Y.Z., Z.C.) declare that they have no known competing financial interests or personal relationships that could have appeared to influence the work reported in this article.

## FUNDING

This study was supported by the Natural Science Foundation of Guangdong Province (2022A1515011714).
